# Mental and Physical Suffering

**Published:** 1875-07

**Authors:** 


### MENTAL AND PHYSICAL SUFFERING.

The sources and uses of suffering may be sought. We are accustomed to generalize too much. What is true of certain specific kinds of suffering is not true of all kinds, moral character is so different. Man is constituted with a tendency toward suffering by birthright. The sins of the father and mother are visited upon the child —not technical sin as we understand it, but certain habits and tendencies are transmitted. If a child comes into the world with a malformation, you can never make a gymnast of him. If he is deficient in intellect he will never stand on a par with other men. If a man be born so that his heritable composition is abnormal, he will suffer, not on account of any sin of his own, but on account of the great law that he carries over the sins and the good and bad qualities of one generation to another—which is, in other words, the motive for right living and the plea for good living. He that corrupts his physical frame, or his moral constitution, transmits the consequent evils down the generations. He that manhoods himself becomes a benefactor of nations yet unborn. His manhood becomes in an organic sense, transmissible. Who has sinned—this man or his parents— that he was born blind? Neither, but that the works of God might be made manifest. Any fair analysis must take into consideration that persons are not responsible for their sufferings, but they are responsible for what uses they put them to. We have noticed that all suffering lies in the nervous system. The muscles do not suffer, except through the nerves that run through them; the veins do not suffer; it is the nerves that encircle their outer surface. Without nerves there would be no more feeling than exists in a tortoise shell. There are different qualities of nerves. Some are born with nervous, morbid qualities, and all things that fall upon them make them suffer, while others are so well constituted that those things that are excruciating to the first class are food to them. We are to discriminate between the persons of the first class and the others. So one great cause of suffering is the construction into which persons are commonly born. A second cause is our own voluntary actions. This includes the large amount of bodily suffering that comes from violating the moral laws on account of pride, vanity, selfishness, or kindred reasons. Many of our most acute sufferings if pride would go down, would cease. Pride will throw a pall over the universe to those who indulge in it. This missusing of one's faculties and not rightly using the world outside and in, is what we call the sphere of voluntary suffering. The third kind of suffering is that which is inflicted upon us, other than hereditary suffering. This arise first from a man's social connections. The most genuine of sufferings is that felt among amiable persons, which naturally springs from their social liabilities. It is not safe to have some friends. It involves a double liability. You suffer not only for yourself but for him. He is not a friend who fans us in the Summer time, and draws just as much wind, with each backward motion, to himself as he wafts to us. He is a friend who wraps himself into us; who stands by us gladly in joy, and in trouble more gladly. The man who stands by us in joy and abandons us in sorrow, we look upon as a parasite. You can't marry and have children without making yourself liable to the cares and burdens of the household, and if bankruptcy come, you have added to your personal sorrow grief for the inconveniences that will come to those you love, your wife and little ones. So a man, not only out of his own joy or sorrow, weeps and rejoices, but out of the weeping or rejoicing of those about him. Those sufferings are by far the largest and noblest of any so far mentioned, on account of their connection with other people. In this world, where so seldom are all friends wise and where so many are imperfect, those who attempt to carry each other's burdens are liable to a great amont of suffering. Civil organization—that which belongs to kingdoms and that which belongs to commonwealths—has its dividend of suffering in each man's experience. Thus in the great war that spread its murky shadow over all this broad land how many thousand, and hundreds of thousands suffered grievously through no fault of their own, but in participation with the commonwealth! The commonwealth treasures you up; every day it is silently pouring innumerable blessings upon your heads, but now and then every citizen has to take his share, his dividend if I may call it so, of suffering on account of the commonwealth. The suffering of the war was not confined to the dead of the battle-field. The drops that fell on the hearthstone spoke of worse suffering than the drops of blood that were shed. Not they that fell haply in the defense of the Union and became glorified by their sacrifice in behalf of duty, suffered alone. They who lived suffered more through their social and civic relations.

---

Avoid Gossip.—We condemn gossip—scandal's twin sister—yet it is a fault easily committed. We begin by a gentle deprecatory reference to somebody's infirmity of temper, and we find ourselves specifying a particular time and scene, which straightway the one who hears tells again to some one else with additions, slight, perhaps, but material. Before we know it we have stirred up a hornet's nest. This may be done without any more potent motive than a mere love of fun—and half the gossip in the social world is of the unthinking kind, indulged in merely from a spirit of drollery. Far worse is that other sort of talk which ends in slander and begins in malice, and which separates friends and sunders the ties of years of intercourse with its sharp and jarring discords. The only way to avoid the evil is to refrain from making the affairs of our friends a staple article of conversation in the household. There are plenty of subjects at hand—let us avoid personalities.

---

Nothing is intolerable that is neccessary.

